# Tetra­aqua­bis­(2-methyl-1*H*-benzimidazolium-1,3-diacetato-κ*O*)cobalt(II) tetra­hydrate

**DOI:** 10.1107/S1600536811029059

**Published:** 2011-08-02

**Authors:** Xiu-Ling Feng, Yu-Ping Zhang

**Affiliations:** a College of Chemistry and Chemical Engineering, Huaihua University, Huaihua 418008, People’s Republic of China; b Wuling Electric Power Group Corporation, Changsha 410000, People’s Republic of China

## Abstract

In the title compound, [Co(C_12_H_11_N_2_O_4_)_2_(H_2_O)_4_]·4H_2_O, the Co^II^ atom lies on an inversion center and is octa­hedrally coordinated by six O atoms from four water mol­ecules and two monodentate zwitterionic 2-methyl­benzimidazolium-1,3-diacetate ligands. An intra­molecular O—H⋯O hydrogen bond occurs. In the crystal, inter­molecular O—H⋯O hydrogen bonds link the mol­ecules into a three-dimensional network. π–π inter­actions between the imidazole and benzene rings [centroid–centroid distance = 3.9031 (17) Å] consolidate the crystal packing.

## Related literature

For general background to coordination polymers, see: Kitagawa *et al.* (2004 ▶); Robson (2000 ▶). For a related structure, see: Lian *et al.* (2009 ▶).
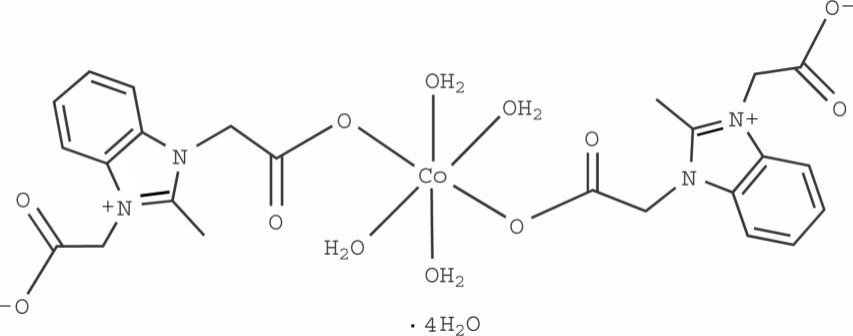


## Experimental

### 

#### Crystal data

[Co(C_12_H_11_N_2_O_4_)_2_(H_2_O)_4_]·4H_2_O
*M* *_r_* = 697.51
Monoclinic, 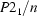

*a* = 7.2930 (7) Å
*b* = 21.240 (2) Å
*c* = 9.8123 (11) Å
β = 104.907 (1)°
*V* = 1468.8 (3) Å^3^
*Z* = 2
Mo *K*α radiation
μ = 0.67 mm^−1^
*T* = 298 K
0.30 × 0.21 × 0.14 mm

#### Data collection

Bruker APEX CCD diffractometer
Absorption correction: multi-scan (*SADABS*; Sheldrick, 1996 ▶) *T*_min_ = 0.825, *T*_max_ = 0.912
7434 measured reflections
2594 independent reflections
1922 reflections with *I* > 2σ(*I*)
*R*_int_ = 0.038

#### Refinement

*R*[*F* ^2^ > 2σ(*F* ^2^)] = 0.041
*wR*(*F* ^2^) = 0.103
*S* = 0.96
2594 reflections
205 parameters
H-atom parameters constrained
Δρ_max_ = 1.03 e Å^−3^
Δρ_min_ = −0.29 e Å^−3^

### 

Data collection: *SMART* (Bruker, 2007 ▶); cell refinement: *SAINT* (Bruker, 2007 ▶); data reduction: *SAINT*; program(s) used to solve structure: *SHELXS97* (Sheldrick, 2008 ▶); program(s) used to refine structure: *SHELXL97* (Sheldrick, 2008 ▶); molecular graphics: *SHELXTL* (Sheldrick, 2008 ▶); software used to prepare material for publication: *SHELXTL*.

## Supplementary Material

Crystal structure: contains datablock(s) I, global. DOI: 10.1107/S1600536811029059/hy2449sup1.cif

Structure factors: contains datablock(s) I. DOI: 10.1107/S1600536811029059/hy2449Isup2.hkl

Additional supplementary materials:  crystallographic information; 3D view; checkCIF report

## Figures and Tables

**Table 1:** Hydrogen-bond geometry (Å, °)

*D*—H⋯*A*	*D*—H	H⋯*A*	*D*⋯*A*	*D*—H⋯*A*
O5—H5*D*⋯O4^i^	0.85	1.89	2.742 (3)	180
O5—H5*E*⋯O7^ii^	0.85	1.86	2.706 (3)	179
O6—H6*C*⋯O4^iii^	0.85	2.05	2.847 (3)	156
O6—H6*D*⋯O2	0.85	1.85	2.614 (2)	149
O7—H7*C*⋯O1^iv^	0.85	2.02	2.859 (3)	170
O7—H7*D*⋯O8^v^	0.85	1.96	2.797 (3)	170
O8—H8*F*⋯O4^i^	0.85	1.96	2.793 (3)	168
O8—H8*G*⋯O3^vi^	0.85	2.03	2.863 (3)	169

Symmetry codes: (i) 
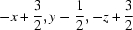
; (ii) 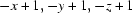; (iii) 
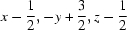
; (iv) 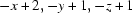; (v) 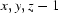; (vi) 
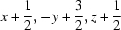
.

**Fig. 1.**
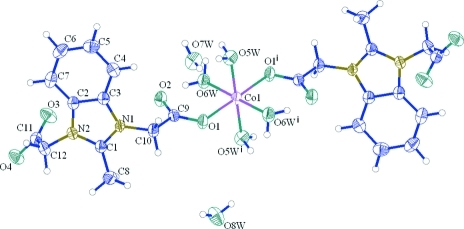
The molecular structure of the title compound. Displacement ellipsoids are drawn at the 50% probability level. [Symmetry code: (i) 1-x, 1-y, 1-z.]

**Fig. 2.**
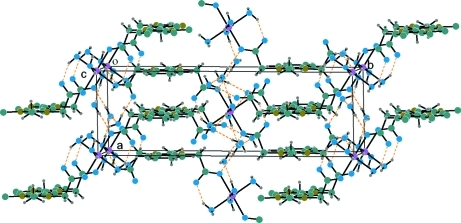
The packing diagram of the title compound. Hydrogen bonds are shown as dashed lines.

## supplementary crystallographic information

### Comment

Coordination polymers have attracted much interest due to their potential applications in many areas such as catalysis, molecular adsorption, magnetism properties and non-linear optics (Kitagawa *et al.*, 2004; Robson, 2000). We report herein the structure of the title compound based on a flexible ligand 1-acetoxy-2-methylbenzimidazole-3-acetate acid (Lian *et al.*, 2009).

The asymmetric unit of the title compound (Fig. 1) contains half of the complex molecule and two uncoordinated water molecules. The Co^II^ atom lies on an inversion center and is octahedrally coordinated by six O atoms from four water molecules and two 2-methylbenzimidazolium-1,3-diacetate ligands. In the crystal structure, intra- and intermolecular O—H···O hydrogen bonds link the molecules into a three-dimensional network (Fig. 2). π–π interactions between the imidazole and benzene rings [centroid–centroid distance = 3.9031 (17) Å] consolidate the crystal packing.

### Experimental

A methanol solution (6 ml) of Co(CH_3_COO)_2_.4H_2_O (0.6 mmol) was slowly added to an aqueous solution (8 ml) of 1-acetoxy-2-methylbenzimidazole-3-acetate acid (0.4 mmol) at room temperature. Red block crystals were obtained after two months in 30% yield based on Co.

### Refinement

H atoms of water molecules were located in difference Fourier maps and refined as riding atoms, with a distance restraint of O—H = 0.85 Å and *U*_iso_(H) = 1.2*U*_eq_(O). H atoms bonded to C atoms were positioned geometrically and refined as riding atoms, with C—H = 0.93 (aromatic), 0.97 (methylene) and 0.96 (methyl) Å and with *U*_iso_(H) = 1.2(1.5 for methyl)*U*_eq_(C). The highest residual electron density was found at 1.43 Å from H8F atom and the deepest hole at 0.80 Å from Co1 atom.

### Crystal data

**Table:**

[Co(C_12_H_11_N_2_O_4_)_2_(H_2_O)_4_]·4H_2_O	*F*(000) = 730
*M**_r_* = 697.51	*D*_x_ = 1.577 Mg m^−^^3^
Monoclinic, *P*2_1_/*n*	Mo *K*α radiation, λ = 0.71073 Å
Hall symbol: -P 2yn	Cell parameters from 2105 reflections
*a* = 7.2930 (7) Å	θ = 2.9–24.1°
*b* = 21.240 (2) Å	µ = 0.67 mm^−^^1^
*c* = 9.8123 (11) Å	*T* = 298 K
β = 104.907 (1)°	Block, red
*V* = 1468.8 (3) Å^3^	0.30 × 0.21 × 0.14 mm
*Z* = 2	

### Data collection

**Table:**

Bruker APEX CCD diffractometer	2594 independent reflections
Radiation source: fine-focus sealed tube	1922 reflections with *I* > 2σ(*I*)
graphite	*R*_int_ = 0.038
φ and ω scans	θ_max_ = 25.0°, θ_min_ = 2.4°
Absorption correction: multi-scan (*SADABS*; Sheldrick, 1996)	*h* = −8→8
*T*_min_ = 0.825, *T*_max_ = 0.912	*k* = −22→25
7434 measured reflections	*l* = −9→11

### Refinement

**Table:**

Refinement on *F*^2^	Primary atom site location: structure-invariant direct methods
Least-squares matrix: full	Secondary atom site location: difference Fourier map
*R*[*F*^2^ > 2σ(*F*^2^)] = 0.041	Hydrogen site location: inferred from neighbouring sites
*wR*(*F*^2^) = 0.103	H-atom parameters constrained
*S* = 0.96	*w* = 1/[σ^2^(*F*_o_^2^) + (0.0577*P*)^2^] where *P* = (*F*_o_^2^ + 2*F*_c_^2^)/3
2594 reflections	(Δ/σ)_max_ < 0.001
205 parameters	Δρ_max_ = 1.03 e Å^−^^3^
0 restraints	Δρ_min_ = −0.29 e Å^−^^3^

### Fractional atomic coordinates and isotropic or equivalent isotropic displacement parameters (Å^2^)

**Table:**

	*x*	*y*	*z*	*U*_iso_*/*U*_eq_	
Co1	0.5000	0.5000	0.5000	0.02777 (17)	
N1	0.9890 (3)	0.69581 (10)	0.5346 (2)	0.0260 (5)	
N2	0.9847 (3)	0.79831 (10)	0.5396 (2)	0.0271 (5)	
O1	0.7555 (2)	0.55086 (8)	0.57519 (19)	0.0330 (5)	
O2	0.6575 (2)	0.63795 (9)	0.4517 (2)	0.0360 (5)	
O3	0.6708 (3)	0.87653 (10)	0.4610 (2)	0.0436 (5)	
O4	0.8026 (3)	0.94740 (9)	0.6224 (2)	0.0420 (5)	
O5	0.4298 (3)	0.51633 (9)	0.6890 (2)	0.0378 (5)	
H5D	0.5133	0.4951	0.7474	0.045*	
H5E	0.3210	0.5052	0.6967	0.045*	
O6	0.3338 (2)	0.57873 (8)	0.4121 (2)	0.0362 (5)	
H6C	0.2891	0.5705	0.3251	0.043*	
H6D	0.4099	0.6094	0.4164	0.043*	
O7	0.9160 (3)	0.52003 (10)	0.2867 (2)	0.0486 (6)	
H7C	1.0178	0.4993	0.3180	0.058*	
H7D	0.9076	0.5300	0.2014	0.058*	
O8	0.8449 (3)	0.56024 (10)	1.0065 (2)	0.0514 (6)	
H8F	0.8127	0.5261	0.9615	0.062*	
H8G	0.9424	0.5748	0.9852	0.062*	
C1	0.9848 (4)	0.74613 (12)	0.6159 (3)	0.0261 (6)	
C2	0.9827 (4)	0.78113 (12)	0.4026 (3)	0.0270 (6)	
C3	0.9845 (4)	0.71627 (12)	0.3987 (3)	0.0270 (6)	
C4	0.9742 (4)	0.68298 (14)	0.2765 (3)	0.0334 (7)	
H4	0.9752	0.6392	0.2748	0.040*	
C5	0.9624 (4)	0.71819 (15)	0.1572 (3)	0.0391 (7)	
H5A	0.9547	0.6978	0.0721	0.047*	
C6	0.9615 (4)	0.78393 (15)	0.1605 (3)	0.0411 (8)	
H6A	0.9532	0.8061	0.0773	0.049*	
C7	0.9727 (4)	0.81696 (14)	0.2830 (3)	0.0367 (7)	
H7	0.9734	0.8607	0.2853	0.044*	
C8	0.9780 (4)	0.74391 (14)	0.7636 (3)	0.0373 (7)	
H8A	1.0481	0.7787	0.8138	0.056*	
H8B	1.0329	0.7051	0.8054	0.056*	
H8C	0.8484	0.7464	0.7686	0.056*	
C9	0.7804 (4)	0.60450 (12)	0.5301 (3)	0.0259 (6)	
C10	0.9814 (4)	0.63010 (12)	0.5744 (3)	0.0294 (6)	
H10A	1.0296	0.6261	0.6758	0.035*	
H10B	1.0621	0.6054	0.5303	0.035*	
C11	0.8025 (4)	0.89776 (13)	0.5524 (3)	0.0303 (6)	
C12	0.9927 (4)	0.86308 (12)	0.5904 (3)	0.0319 (6)	
H12A	1.0823	0.8864	0.5519	0.038*	
H12B	1.0409	0.8628	0.6922	0.038*	

### Atomic displacement parameters (Å^2^)

**Table:**

	*U*^11^	*U*^22^	*U*^33^	*U*^12^	*U*^13^	*U*^23^
Co1	0.0247 (3)	0.0239 (3)	0.0339 (3)	−0.0009 (2)	0.0062 (2)	0.0047 (2)
N1	0.0230 (11)	0.0213 (12)	0.0321 (13)	−0.0029 (9)	0.0042 (10)	0.0030 (9)
N2	0.0252 (12)	0.0210 (12)	0.0336 (13)	−0.0013 (9)	0.0049 (10)	−0.0016 (9)
O1	0.0307 (10)	0.0249 (11)	0.0408 (11)	−0.0050 (8)	0.0043 (9)	0.0085 (8)
O2	0.0251 (10)	0.0307 (11)	0.0479 (12)	−0.0007 (9)	0.0017 (9)	0.0102 (9)
O3	0.0330 (11)	0.0439 (13)	0.0475 (13)	0.0033 (10)	−0.0014 (10)	−0.0140 (10)
O4	0.0455 (12)	0.0305 (12)	0.0453 (13)	0.0092 (9)	0.0030 (10)	−0.0110 (9)
O5	0.0300 (11)	0.0438 (13)	0.0394 (12)	0.0004 (9)	0.0090 (9)	0.0057 (9)
O6	0.0323 (11)	0.0297 (11)	0.0431 (12)	−0.0013 (9)	0.0035 (9)	0.0052 (8)
O7	0.0363 (12)	0.0547 (14)	0.0568 (14)	0.0068 (10)	0.0155 (11)	0.0069 (11)
O8	0.0426 (13)	0.0466 (14)	0.0690 (16)	−0.0077 (11)	0.0219 (11)	−0.0166 (11)
C1	0.0209 (14)	0.0255 (15)	0.0300 (15)	−0.0009 (11)	0.0032 (11)	0.0014 (11)
C2	0.0225 (14)	0.0250 (15)	0.0339 (15)	0.0003 (11)	0.0079 (11)	0.0017 (11)
C3	0.0208 (14)	0.0271 (15)	0.0327 (15)	−0.0001 (11)	0.0062 (11)	0.0013 (11)
C4	0.0291 (15)	0.0314 (17)	0.0403 (17)	0.0002 (12)	0.0096 (13)	−0.0041 (13)
C5	0.0359 (17)	0.050 (2)	0.0338 (17)	0.0012 (14)	0.0128 (13)	−0.0035 (14)
C6	0.0379 (18)	0.051 (2)	0.0366 (18)	0.0008 (15)	0.0137 (14)	0.0141 (14)
C7	0.0329 (16)	0.0305 (17)	0.0480 (19)	0.0010 (13)	0.0126 (14)	0.0099 (13)
C8	0.0368 (17)	0.0397 (18)	0.0335 (17)	−0.0052 (13)	0.0058 (13)	0.0014 (13)
C9	0.0296 (15)	0.0227 (15)	0.0274 (14)	−0.0018 (12)	0.0108 (12)	−0.0013 (11)
C10	0.0267 (15)	0.0223 (15)	0.0367 (16)	−0.0007 (11)	0.0039 (12)	0.0035 (11)
C11	0.0326 (16)	0.0272 (16)	0.0309 (16)	−0.0010 (12)	0.0078 (13)	0.0033 (12)
C12	0.0281 (15)	0.0239 (15)	0.0421 (17)	−0.0015 (12)	0.0059 (12)	−0.0047 (12)

### Geometric parameters (Å, °)

**Table:**

Co1—O5	2.0758 (19)	O8—H8F	0.8500
Co1—O5^i^	2.0758 (19)	O8—H8G	0.8501
Co1—O6^i^	2.1146 (17)	C1—C8	1.464 (4)
Co1—O6	2.1146 (17)	C2—C3	1.378 (4)
Co1—O1	2.1157 (17)	C2—C7	1.384 (4)
Co1—O1^i^	2.1157 (17)	C3—C4	1.377 (4)
N1—C1	1.339 (3)	C4—C5	1.373 (4)
N1—C3	1.395 (3)	C4—H4	0.9300
N1—C10	1.454 (3)	C5—C6	1.397 (4)
N2—C1	1.338 (3)	C5—H5A	0.9300
N2—C2	1.390 (3)	C6—C7	1.376 (4)
N2—C12	1.459 (3)	C6—H6A	0.9300
O1—C9	1.252 (3)	C7—H7	0.9300
O2—C9	1.243 (3)	C8—H8A	0.9600
O3—C11	1.219 (3)	C8—H8B	0.9600
O4—C11	1.258 (3)	C8—H8C	0.9600
O5—H5D	0.8500	C9—C10	1.518 (3)
O5—H5E	0.8500	C10—H10A	0.9700
O6—H6C	0.8500	C10—H10B	0.9700
O6—H6D	0.8500	C11—C12	1.530 (4)
O7—H7C	0.8500	C12—H12A	0.9700
O7—H7D	0.8501	C12—H12B	0.9700
			
O5—Co1—O5^i^	180.0	C2—C3—N1	106.4 (2)
O5—Co1—O6^i^	90.84 (7)	C5—C4—C3	116.1 (3)
O5^i^—Co1—O6^i^	89.16 (8)	C5—C4—H4	122.0
O5—Co1—O6	89.16 (8)	C3—C4—H4	122.0
O5^i^—Co1—O6	90.84 (7)	C4—C5—C6	121.6 (3)
O6^i^—Co1—O6	180.0	C4—C5—H5A	119.2
O5—Co1—O1	90.03 (8)	C6—C5—H5A	119.2
O5^i^—Co1—O1	89.97 (8)	C7—C6—C5	122.0 (3)
O6^i^—Co1—O1	84.29 (7)	C7—C6—H6A	119.0
O6—Co1—O1	95.71 (7)	C5—C6—H6A	119.0
O5—Co1—O1^i^	89.97 (8)	C6—C7—C2	116.0 (3)
O5^i^—Co1—O1^i^	90.03 (8)	C6—C7—H7	122.0
O6^i^—Co1—O1^i^	95.71 (7)	C2—C7—H7	122.0
O6—Co1—O1^i^	84.29 (7)	C1—C8—H8A	109.5
O1—Co1—O1^i^	180.00 (8)	C1—C8—H8B	109.5
C1—N1—C3	108.8 (2)	H8A—C8—H8B	109.5
C1—N1—C10	126.7 (2)	C1—C8—H8C	109.5
C3—N1—C10	124.2 (2)	H8A—C8—H8C	109.5
C1—N2—C2	108.8 (2)	H8B—C8—H8C	109.5
C1—N2—C12	126.5 (2)	O2—C9—O1	126.3 (2)
C2—N2—C12	124.6 (2)	O2—C9—C10	117.5 (2)
C9—O1—Co1	122.42 (16)	O1—C9—C10	116.2 (2)
Co1—O5—H5D	102.5	N1—C10—C9	111.6 (2)
Co1—O5—H5E	118.9	N1—C10—H10A	109.3
H5D—O5—H5E	108.4	C9—C10—H10A	109.3
Co1—O6—H6C	106.2	N1—C10—H10B	109.3
Co1—O6—H6D	106.7	C9—C10—H10B	109.3
H6C—O6—H6D	106.5	H10A—C10—H10B	108.0
H7C—O7—H7D	108.6	O3—C11—O4	127.0 (3)
H8F—O8—H8G	108.7	O3—C11—C12	119.6 (2)
N2—C1—N1	108.9 (2)	O4—C11—C12	113.4 (2)
N2—C1—C8	125.9 (2)	N2—C12—C11	114.6 (2)
N1—C1—C8	125.2 (2)	N2—C12—H12A	108.6
C3—C2—C7	121.7 (3)	C11—C12—H12A	108.6
C3—C2—N2	106.9 (2)	N2—C12—H12B	108.6
C7—C2—N2	131.4 (3)	C11—C12—H12B	108.6
C4—C3—C2	122.6 (2)	H12A—C12—H12B	107.6
C4—C3—N1	130.9 (3)		
			
O5—Co1—O1—C9	−107.6 (2)	C10—N1—C3—C4	−0.6 (4)
O5^i^—Co1—O1—C9	72.4 (2)	C1—N1—C3—C2	1.6 (3)
O6^i^—Co1—O1—C9	161.6 (2)	C10—N1—C3—C2	176.4 (2)
O6—Co1—O1—C9	−18.4 (2)	C2—C3—C4—C5	0.1 (4)
C2—N2—C1—N1	1.9 (3)	N1—C3—C4—C5	176.7 (3)
C12—N2—C1—N1	−175.9 (2)	C3—C4—C5—C6	0.2 (4)
C2—N2—C1—C8	−177.1 (3)	C4—C5—C6—C7	0.1 (4)
C12—N2—C1—C8	5.1 (4)	C5—C6—C7—C2	−0.7 (4)
C3—N1—C1—N2	−2.2 (3)	C3—C2—C7—C6	1.0 (4)
C10—N1—C1—N2	−176.8 (2)	N2—C2—C7—C6	−176.0 (3)
C3—N1—C1—C8	176.8 (2)	Co1—O1—C9—O2	9.4 (4)
C10—N1—C1—C8	2.2 (4)	Co1—O1—C9—C10	−169.50 (17)
C1—N2—C2—C3	−0.9 (3)	C1—N1—C10—C9	95.3 (3)
C12—N2—C2—C3	177.0 (2)	C3—N1—C10—C9	−78.6 (3)
C1—N2—C2—C7	176.4 (3)	O2—C9—C10—N1	9.7 (3)
C12—N2—C2—C7	−5.7 (4)	O1—C9—C10—N1	−171.3 (2)
C7—C2—C3—C4	−0.8 (4)	C1—N2—C12—C11	−102.4 (3)
N2—C2—C3—C4	176.8 (2)	C2—N2—C12—C11	80.1 (3)
C7—C2—C3—N1	−178.1 (2)	O3—C11—C12—N2	−15.7 (4)
N2—C2—C3—N1	−0.5 (3)	O4—C11—C12—N2	164.9 (2)
C1—N1—C3—C4	−175.4 (3)		

Symmetry codes: (i) −*x*+1, −*y*+1, −*z*+1.

### Hydrogen-bond geometry (Å, °)

**Table:**

*D*—H···*A*	*D*—H	H···*A*	*D*···*A*	*D*—H···*A*
O5—H5D···O4^ii^	0.85	1.89	2.742 (3)	180
O5—H5E···O7^i^	0.85	1.86	2.706 (3)	179
O6—H6C···O4^iii^	0.85	2.05	2.847 (3)	156
O6—H6D···O2	0.85	1.85	2.614 (2)	149
O7—H7C···O1^iv^	0.85	2.02	2.859 (3)	170
O7—H7D···O8^v^	0.85	1.96	2.797 (3)	170
O8—H8F···O4^ii^	0.85	1.96	2.793 (3)	168
O8—H8G···O3^vi^	0.85	2.03	2.863 (3)	169

Symmetry codes: (ii) −*x*+3/2, *y*−1/2, −*z*+3/2; (i) −*x*+1, −*y*+1, −*z*+1; (iii) *x*−1/2, −*y*+3/2, *z*−1/2; (iv) −*x*+2, −*y*+1, −*z*+1; (v) *x*, *y*, *z*−1; (vi) *x*+1/2, −*y*+3/2, *z*+1/2.

## References

[bb1] Bruker (2007). *SMART* and *SAINT* Bruker AXS Inc., Madison, Wisconsin, USA.

[bb2] Kitagawa, S., Kitaura, R. & Noro, S. (2004). *Angew. Chem. Int. Ed.* **43**, 2334–2375. 10.1002/anie.200300610 15114565

[bb3] Lian, H.-C., Ni, Q.-L., Jiang, X.-F., Cen, Z.-M. & Lin, J.-H. (2009). *Acta Cryst.* E**65**, m1091. 10.1107/S1600536809031766 PMC2969862 21577442

[bb4] Robson, R. (2000). *J. Chem. Soc. Dalton Trans.* pp. 3735–3744.

[bb5] Sheldrick, G. M. (1996). *SADABS* University of Göttingen, Germany.

[bb6] Sheldrick, G. M. (2008). *Acta Cryst.* A**64**, 112–122. 10.1107/S0108767307043930 18156677

